# Characterization and evaluation of a *Sarcoptes scabiei* allergen as a candidate vaccine

**DOI:** 10.1186/1756-3305-5-176

**Published:** 2012-08-16

**Authors:** Runhui Zhang, Quwu Jise, Wanpeng Zheng, Yongjun Ren, Xiang Nong, Xuhang Wu, Xiaobin Gu, Shuxian Wang, Xuerong Peng, Songjia Lai, Guangyou Yang

**Affiliations:** 1 Department of Parasitology, College of Veterinary Medicine, Sichuan Agricultural University, Ya’an, 625014, China; 2 Sichuan Academy of Animal Husbandry Sciences, Chengdu, 610066, China; 3 Department of Chemistry, College of Life and Basic Science, Sichuan Agricultural University, Ya’an, 625014, China; 4 College of Animal Science and Technology, Sichuan Agricultural University, Ya’an, 625014, China

**Keywords:** *Sarcoptes scabiei*, Tropomyosin, Immunolocalization, Vaccine

## Abstract

**Background:**

Sarcoptic mange caused by the mite *Sarcoptes scabiei* is a worldwide disease affecting both humans and animals. Here we report the molecular characterization and evaluation of a recombinant *S. scabiei* tropomyosin (SsTm) protein in a vaccination trial in rabbits.

**Methods:**

The full-length cDNA was cloned in a bacterial pET vector, and the recombinant protein was expressed in BL21 (DE3) cells and purified. Using specific rabbit antiserum, tropomyosin was localized immunohistochemically in mite tissue sections. Vaccination trials with the recombiant SsTm was carried out in New Zealand rabbits.

**Results:**

The full-length open reading frame (ORF) of the 852 bp cloned gene from *S. scabiei* encodes a 32.9 kDa protein. The amino acid sequence showed 98.94%, 97.89% and 98.59% homology to *Dermatophagoides farina* and *Dermatophagoides pteronyssinus* group 10 allergens and *Psoroptes ovis* tropomyosin, respectively. Tropomyosin was localized immunohistochemically in mite tissue sections mainly in the mouthparts, legs and integument of the epidermis. The predicted cross-reactivity of SsTm indicated that it is an allergenic protein. While vaccination with the recombiant SsTm resulted in high levels of specific IgG (*P* < 0.01), a low IgE antibody response and no significant protection against *S. scabiei* challenge were observed. After challenge, specific IgG levels remained significantly higher than the control (*P* < 0.01), while changes of total IgE levels were not significant (*P* > 0.05). However, the lesion areas in the vaccination group decreased at the end of the experiment compared with controls.

**Conclusions:**

Although vaccination with recombinant SsTm did not efficiently control sarcoptic mange in rabbits, the immunogenic properties of tropomyosin suggest it may be developed as a vaccine with alternative adjuvants or delivery methods.

## Background

Sarcoptic mange, caused by ectoparasite infestation with the mite *Sarcoptes scabiei*, is a disease distributed worldwide in both humans and animals. Overcrowded living conditions, poverty and poor hygiene are significant factors [1] for infection with *S. scabiei* mites in the currently estimated 300 million people worldwide [2,3]. In addition, *Sarcoptes* are common ectoparasites in domestic and wild populations of canids, cats, ungulates, boars, wombats, koalas, great apes and bovids [4]. In Spain, sarcoptic mange is a widespread disease in wild rabbits [5]. The sarcoptes mites burrow into the epidermis and lay eggs in the stratum corneum for weeks, leading to a host immune response and antibody production and resulting in papules on the surface of the skin and pruritus. Sarcoptic mange, if left untreated, may cause significant morbidity and economic losses in livestock. Moreover, high costs are associated with acaricides used in infested livestock [6,7].

Although various acaricides considered as appropriate treatments are generally used to control sarcoptic mange[8], they can be highly toxic and strong resistance to them can be developed. Furthermore, the quality and safety of livestock products are threatened with the potential for accidental environmental pollution with acaricides [9]. Thus, the Sarcoptes World Molecular Network (WMN) [10] was established to coordinate and support additional epidemiological, diagnostic, treatment and molecular studies of scabies mites.

Tropomyosin, a microfilament protein with a ɑ-helical coiled-coil structure, is found in all cell types [11]. It has been identified as a conserved and cross-reactive allergen between mites and other invertebrates [12]. Therefore, tropomyosin is considered as a good model to study the contribution of the primary structure to the allergenicity of proteins [13]. Previous studies have shown that the group 10 tropomyosin allergen of house dust mites induces cross-reactivity of IgE and reacts with specific IgE from humans allergic to these allergens [14]. In addition, the tropomyosin allergen is present in shrimp, mites and insects, and may serve as a potential vaccine candidate antigen along with myosin and paramyosin [15], [16]. Successful vaccination with recombinant tropomysin has been reported in other parasites. For instance, immunization with a tropomyosin-like protein purified from the rodent filarial *Acanthocheilonema viteae* was shown to decrease the number of adult worm burden by up to 65% as well as the circulating microfilariae by up to 93% in jirds (*Meriones unguiculatus*) [17]. Furthermore, tryopomyosin is confirmed as an immunodominant allergen of sheep scab mites and shows promise as a vaccine candidate [18,19].

In this study, we isolated the tropomyosin gene of *Sarcoptes scabiei* based on analysis of expressed sequence tags (EST). The recombinant protein (SsTm) was expressed, analyzed and purified for immunization of rabbits. Immunolocalization of tropomyosin in *S. scabiei* tissues was also performed.

## Methods

### Mites and animals

Sarcoptic mites (adults, nymphs and larvae) were collected from rabbits and stored at −70°C prior to RNA extraction. Mites were unfed prior to treatment. Four-month-old naive rabbits were prepared for a vaccination trial at the Laboratory Animal Center of Sichuan Agriculture University (China). All animals were handled in strict accordance with animal protection laws of the People's Republic of China (A draft of an animal protection law in China was released on September 18, 2009). All procedures were strictly carried out according to the Guide for the Care and Use of Laboratory Animals.

### Isolation of total RNA and amplification of cDNA encoding tropomyosin

Extraction of total RNA was carried out using a commercial extraction kit (Waston, Shanghai, China) and transcribed into cDNA using RevertAid^TM^ First Strand cDNA Synthesis Kit (Fermentas) according to the manufacturer’s protocol and stored at −70°C. Based on the *S. scabiei* EST database [20], the foward (5'-ATGGAGGCCATCAAGAAAAAAATG-3') and reverse (5'-TTAATAACCAGTAAGTTCGGCAA-3') primers were used in a reaction mixture containing 5 μL cDNA in a total of 50 μL. The cycling parameters were 94°C for 5 min and 94°C for 45 sec, followed by 30 cycles of 49°C for 45 sec, 72°C for 45 sec and a final extension of 72°C for 10 min. PCR products were separated by agarose gel electrophoresis (2% TAE-agarose gel) and purified with a QIAquick Gel Extraction Kit (Watson) according to the manufacturer’s instructions.

### Expression and purification of recombinant tropomyosin

The full-length coding sequence was amplified by PCR using specific primers (forward primer 5’-CATG* CCATGG *AT*G*GAGGCCATCAAGAAA-3’; reverse primer 5‘-CG* GGATCC *TTAATAACCCATAAGTTC3-’). The PCR products were digested with *Bam*H I and *Nco* I (TaKaRa, Tokyo, Japan) and gel-purified. The cDNA was subcloned into the bacterial expression vector pET-32a (+) (Novagen, Dermstadt, Germany) and used to transform BL21 (DE3) *Escherichia coli* cells (Novagen). Briefly, individual colonies were selected and grown in LB medium with ampicillin (50 μg/ml) at 37°C until the OD_600_ value reached 1.0, and then isopropyl-beta-d-thiogalactopyranoside (IPTG) was added at the final concentration of 1 mM to induce recombinant protein expression for 4 h at 37°C. Purification of the recombinant SsTm was performed as previously described [21].

### Sequence analysis and cross-reactivity prediction

The presence of a signal peptide was detected using SignalP-2.0 at the Center of Biological Sequence Analysis (http://www.cbs.dtu.dk/services/SignalP-2.0/), and cellular localization was predicted using TMHMM (http://www.cbs.dtu.dk/services/TMHMM/). The molecular weight of the predicted protein was calculated using Compute pI/Mw (http://us.expasy.org/tools/pi_tool.html). Cross-reactivity with known allergens was predicted with the web server SDAP (http://fermi.utmb.edu/SDAP/).

### Western blotting

For Western blotting, recombinant SsTm proteins were separated by electrophoresis and subsequently transferred onto a PVDF membrane (Millipore, Dermstadt, Germany) for 1 h in an electrophoretic transfer cell (Bio-Rad, Hercules, California,USA). The membrane was blocked with 5% skimmed milk in TBST (40 mM Tris–HCl, 0.5 M NaCl, 0.1 Tween-20, pH 7.4) for 4 h at room temperature. Subsequently, the membrane was incubated with rabbit antiserum diluted 1:200 (v/v) in 1% skimmed milk-TBST overnight at 4°C. The membrane was washed 3 times (10 min/wash) in TBST and further incubated with alkaline phosphatase-conjugated goat anti-rabbit IgG diluted 1:1000 for 1 h. After another three washes for about 10 min each time with TBST, protein signals were detected using 5-bromo-4-chloro-3'-indolyphosphate (BCIP) and nitro-blue tetrazolium (NBT) substrates.

### Immunolocalization of tropomyosin

In order to perform immunolocalization studies of mite sections, antiserum against SsTm were raised in rabbits using standard procedures [22]. Mites were fixed in 1% molten agarose and set in paraffin wax after solidification of the molten agarose. Briefly, 5 μm sections were cut, dried at 60°C, dewaxed, rehydrated, treated to inactivate endogenous peroxidase and incubated in 25% normal goat serum in TBS for 15 min. Thereafter, the tissue sections were incubated at 4°C overnight with specific rabbit anti-SsTm antibodies diluted 1:1000 in TBS. After the sections were rinsed with TBS, horseradish peroxidase (HRP)-conjugated goat anti-rabbit IgG (AMRESCO, Texas, USA) diluted 1:200 was used for detection of the antibodies and visualized using the EnVision ^TM^ + System, HRP (DAB) (DAKO, Glostrup, Denmark). Sections were counterstained with hematoxylin and rinsed in water and dehydrated. Finally, sections were mounted in a resin-based mountant and viewed with a microscope. Negative controls were carried out by replacement of specific antibodies with negative rabbit serum.

### Vaccination trial

Preparation of the challenge inoculum of *S. scabiei* mites was designed similarly to that described previously [23]. Number of rabbits per group was designed similarly to a study of *S.scabiei* challenge trial [24]. Eighteen female New Zealand rabbits, about 4 months of age, were randomly divided into three groups (6 rabbits per group). Animals were immunized as follows: group 1, 100 μg SsTm mixed with 100 μg QuilA(0.01 g/ml, International Laboratory, USA); group 2, 100 μg QuilA in normal saline (NS) as adjuvant controls; group 3, only NS as unvaccinated controls. All animals were immunized four times by hypodermic injection at 1-week intervals for the first three times and a 2-week interval before the last vaccination. Two weeks after the last vaccination, the rabbits were challenged with approximately 2000 live mites each on the convex surface of the left ear for 48 h maintained by a piece of gauze. Blood samples were taken prior to vaccination, every week during vaccination and challenge until week 4 post-challenge. Skin lesions were photographed weekly after challenge. Mange lesion areas and the body weights were measured during the vaccination trial every week. The lesions were graded as follows: score 0 if no area was infected; score 1 if <10% of the area was infected; score 2 if 10–25% of the area was infected; score 3 if 25–50% of the area was infected; score 4 if 50–100% of the area was infected.

### Enzyme-linked immunosorbent assays (ELISA)

Serum samples were collected from immunized and control rabbits for assessment of antibody responses. Specific IgG and total IgE levels against SsTm were determined by ELISA. Purified SsTm was used to coat 96-well ELISA plates (Invitrogen, Carlsbad, California, USA) at 4 °C overnight in 50 μl of carbonate buffer (0.06 M, pH 9.6). The plates were washed with PBS containing 0.05 % Tween-20 (PBST) four times and then blocked with 100 μl of 1% BSA in PBS and incubated at 37 °C for 1 h. After the wells were washed again with PBST, 100 μl of sera (1:100) were added to the wells and incubated at 37 °C for 1 h. The wells were washed four times with PBST and incubated with HRP-conjugated goat anti-rabbit IgG (1:5000) at 37°C for 1 h. Following the final wash cycle, the wells were incubated with 50 μl TMB substrate (45 mM dibasic sodium phosphate, 0.22 mM citric acid, 0.42 mM 3, 3', 5, 5'tetramethylbenzidine and 30% H_2_O_2_), and the reaction was stopped after 15 min by adding 50 μl per well of 0.5 M H_2_SO_4_. The optical density (OD) was measured at 460 nm using a microplate reader. Total IgE levels were measured using a rabbit IgE ELISA kit (Groundwork biotechnology Diagnosticate, USA) according to the manufacturer’s instructions.

### Statistical analysis

Statistical analysis was performed by analysis of variance (ANOVA), Dunnett’s test and least significant difference (LSD) using SPSS version 13.0 for Windows. Differences with *P-*values < 0.05 were considered statistically significant.

## Results

### Amplification of cDNA encoding tropomyosin

**Figure 1:**
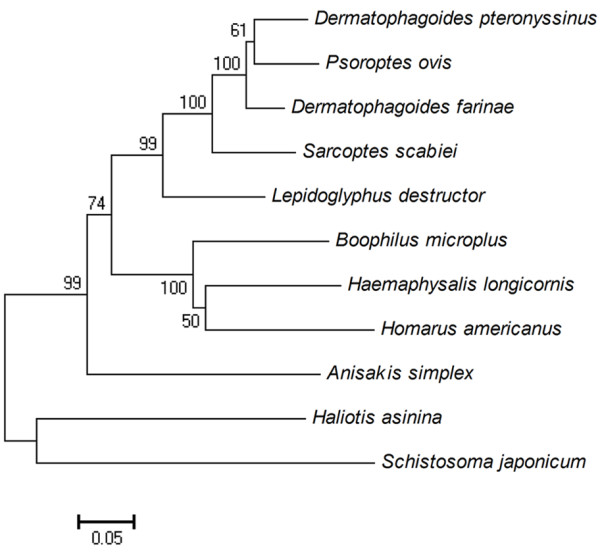
**Neighbor-joining phylogenetic tree of selected tropomyosin proteins.** GenBank accession numbers for each sequence are as follows: *Homarus americanus*, AF034954; *Haemaphysalis longicornis*, AF534184; *Dermatophagoides pteronyssinus*, D17682; *Dermatophagoides gallinae*, AM167555; *Schistosoma japonicum*, L76203; *Boophilus micoplus*, AF124514; *Psoroptes ovis*, AM114276; *Anisakis simplex*, Y19221; *Lepidoglyphus destructor*, AJ250096; *Haliotis asinina*, AY320360; *Sarcoptes scabiei*, JF922117. The percentage of support from 1000 bootstrap replicates is indicated at the nodes. Phylogenetic analysis was conducted using MEGA 4.0 [25].

**Figure 2:**
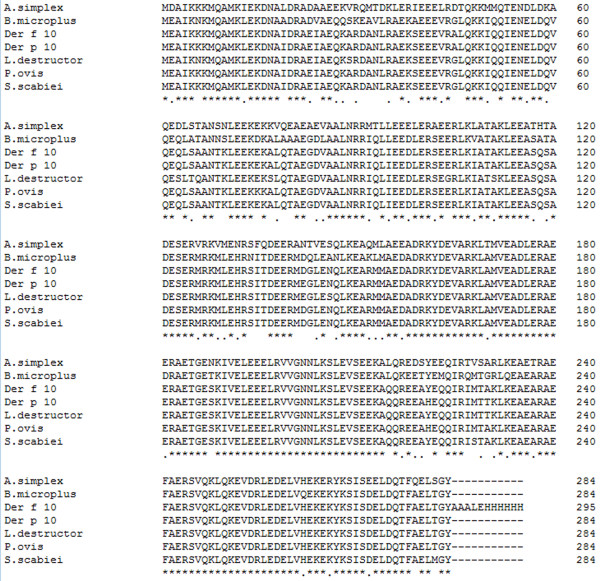
**Comparison of SsTm with the tropomyosin amino acid sequences of other species.** Genebank accession numbers: *A. simplex*, CAB93501; *B. microplus*, AAD17324; *Der f 10*, BAA04557; *Der p 10*, CAJ44440; *L. destructor*, CAB71342; *P. ovis*, CAJ38272; *S. scabiei*, JF922117.

A cDNA sequence was obtained with an open reading frame of 852 bp (GenBank: JF922117) encoding a 284 amino acid tropomyosin protein. By phylogenetic analysis, this tropomyosin sequence from scabies mites showed high homology to that of *Dermatophagoides farinae* (Der f 10) (83.90%), *Dermatophagoides pteronyssinus* (Der p 10) (85.26%), *Psoroptes ovis* (85.03%) and other arthropods (e.g. *Boophilus microplus*, *Haemaphysalis longicornis*, *Lepidoglyphus destructor*) (72%-82%). Other tropomysin sequences in the tree from nematodes, trematodes, fish and ticks all formed separate and distinct clades (Figure 1). The protein was predicted to have a molecular weight of 32.90 kDa, a pI = 4.43, as well as strong hydrophobicity and antigenicity. No signal peptides or transmembrane domains were evident in the protein, indicating that the peptide chain is not anchored to a cellular membrane. An alignment of SsTm with other tropomysin proteins is shown in Figure 2. The amino acid sequence of SsTm is rich in Glu (20.4%), Ala (12.0%) and Lys (9.9%) and highly homologous to Der f 10, Der p 10 and *P. ovis* tropomyosin with identities of 98.94%, 97.89% and 98.59%, respectively.

### Prediction of cross-reactivity

A FASTA alignment was used to identify whether the SsTm protein is potentially cross-reactive based on the Food and Agricultural Organization (FAO)/World Health Organization (WHO) allergenicity rules. The results showed that SsTm is related to 4 allergenic proteins from 4 species of mites (Table 1). The most homologous protein in the Structural Database of Allergenic Proteins (SDAP) to SmTm was found to be *Tyr p* 10 (*Tyrophagus putrescentiae)* from a storage mite (E < 0.01).

**Table 1:** Predicted cross-reactivity between SsTm and known allergens in SDAP

**No**	**Allergen**	**Accession**	**Species**	**Sequence Length**	**bit score**^**a**^	**E score**
1	Tyr p 10.0101	ABQ96644	Storage mite	284	33.9	3.7e-03
2	Der f 10.0101	ABU97468	American house dust mite	289	33.7	4.4e-03
3	Der p 10	CAA75141	European house dust mite	284	33.1	6.4e-03
4	Blo t 10.0101	ABU97466	mite	284	32.9	7.3e-03

^a^The bit score is equivalent to the bit score reported by BLAST. A 1-bit increase in score corresponds to a 2-fold reduction in expectation, and a 10-bit increase implies 1000-fold lower expectation. Sequences with E values < 0.01 are almost always homologous.

### Expression of recombinant SsTm and Western blot analysis

**Figure 3:**
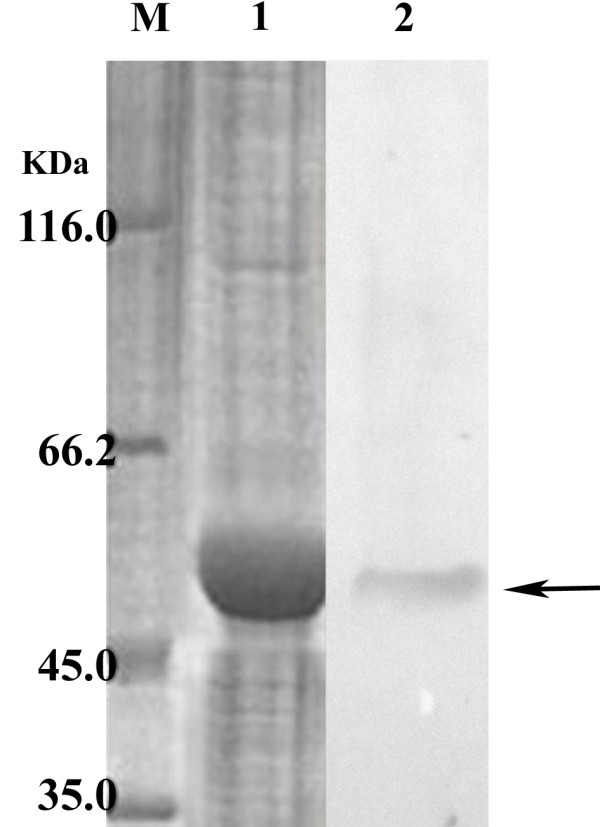
**Western blotting analysis of recombinant proteins.** M, protein marker; lane 1, purified recombinant proteins; lane 2, Western blot results.

SDS-PAGE analysis showed that the molecular weight of the expressed protein was approximately 50 kDa, consisting of the predicted 32.9 kDa SsTm protein and an additional peptide expressed from the pET-32a (+) vector (20 kDa). The recombinant SsTm was confirmed to be a soluble protein. Furthermore, the results showed that the maximum expression of recombinant SsTm was obtained at 37°C at 4 h after induction with 0.4 mol/L IPTG (data not shown). The recombinant SsTm was detected in Western blots by serum raised in rabbits against *S. scabiei* (Figure 3), while no reaction was observed when using the negative control serum (data not shown).

### Immunolocalization

**Figure 4:**
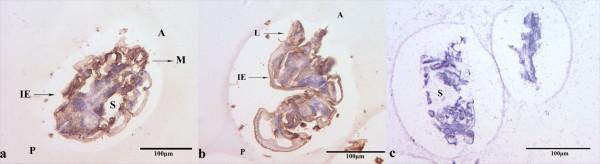
**Immunolocalization of tropomyosin in sections of *S. scabiei.* ** (**A**, **B**) Staining with anti-tropomyosin as primary antibody. (**C**) Control (pre-immune sera). The EnVision TM + System-HRP(DAB) (DAKO) was used, according to the manufacturer’s instructions for detection of the rabbit antibodies. M, mouthparts; L, legs; S, stomach blocks; IE, the integument of epidermis; A, anterior end of mite; P, posterior end of mite.

The staining was evidently widespread in mites, particularly in the mouthparts and legs. The SsTm protein also appeared in the integument of epidermis and stomach muscle (Figure 4). No staining was detected in sections of *S. scabiei* developed with pre-immune serum (data not shown).

### SsTm vaccination trial in rabbits

The protective effects of recombinant SsTm in rabbits against *S. scabiei* was identified by measuring the infested areas (Table 2). Forty-eight hours after challenge, inflammation in almost all of the infected auricles of the rabbits and a number of small papules were observed when the auricles were palpated. No differences were observed among the three groups in lesion levels. Over the course of infection, the papules were replaced by crusts in all rabbits, and the infested areas increased rapidly. At the end of the trial, margins of the infected auricles were covered by thick crusts in some rabbits. Although the mange lesions did not spread to limbs, many mites were detected in skin scrapings from rabbits in the three groups (data not shown). Interestingly, the lesion areas in vaccinated groups were apparently reduced compared with NS control groups at the end of the experiment. However, it was difficult to distinguish between the vaccinated group and controls by identifying the severity of lesions.

**Table 2:** **Mean mange lesion scores**^**a**^** after challenge**

	**prior to challenge**	**week 1**	**week 2**	**week 3**	**week 4**
unvaccinated group	0	2.08±0.39	2.36±0.16	2.7±0.10	3.06±0.10
SsTm group	0	1.96±0.34	2.67±0.32	3±0.26	2.74±0.19
QuilA group	0	2±0.13	2.88±0.24	3.5±0.56	3.68±0.10

^a^The lesions were graded as follows: score 0 if no area was infected; score 1 if <10% of the area was infected; score 2 if 10–25% of the area was infected; score 3 if 25–50% of the area was infected; score 4 if 50–100% of the area was infected.

**Figure 5:**
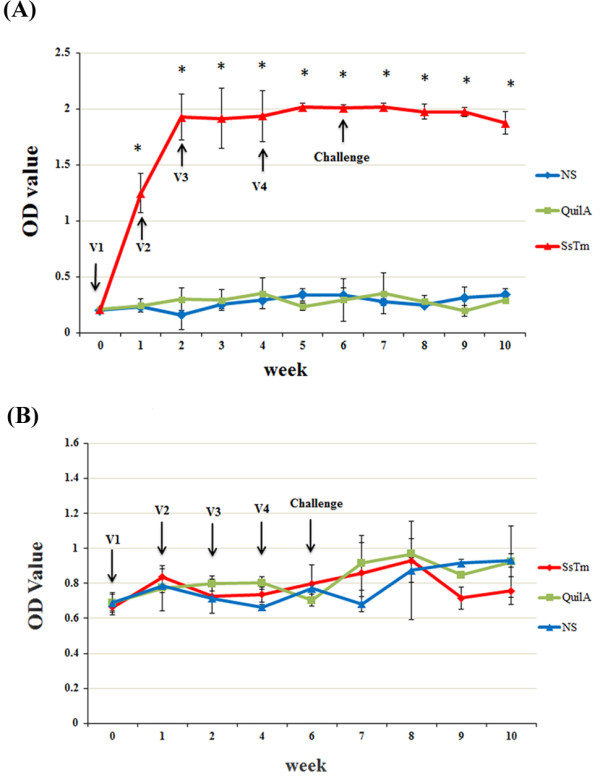
**Specific IgG(A) and IgE(B) antibody levels in sera of rabbits immunized with *S.*  *scabiei* tropomyosin detected by ELISA.** Rabbits were vaccinated four times with SsTm, QuilA and saline and challenged with approximately 2000 mites. Results are shown as means (± S.D.). OD values were determined as absorbance at 450 nm. V1, first vaccination; V2, second vaccination; V3, third vaccination; V4, fourth vaccination. *P < 0.01, compared with NS control group.

Serological responses to SsTm vaccination were evaluated by ELISA (Figures 5). Scabies-specific IgG antibodies increased after the first immunization in all the vaccinated animals (*P* < 0.001), which were significantly higher than the levels observed in QuilA groups and non-vaccinated groups (*P* < 0.001). The levels of scabies-specific IgG antibodies peaked (OD_450_ 2.02) at the fifth week and stabilized at high levels after challenge in the vaccinated groups. In the QuilA and non-vaccinated groups, the OD_450_ values of specific IgG remained low throughout the experiment. Due to the lack of an effective secondary anti-rabbit IgE antibody, we chose to measure the levels of total IgE, which did not change obviously after the four immunizations compared with the levels of pre-immune serum in all animals. In the four weeks after challenge, IgE levels of the unvaccinated group and QuilA group increased slightly, but the OD_450_ value was still low (OD_450_ < 1.0). No statistically significant difference was observed between the SsTm group and saline group (*P* > 0.05). However, specific antibody titers decreased slightly at weeks 9 and 10 and may be associated with the reduced lesion areas in the vaccinated group.

## Discussion

In this study, we described the molecular characterization of tropomyosin from *Sarcoptes scabiei* and evaluated its ability to induce protection against infection in rabbits. An EST study of *S. scabiei* has identified the presence of a sequence with high homology to the Mag44 sequence in dust mites [20]. Here, we successfully cloned and expressed SsTm encoded by the *tropomyosin* gene, and this protein revealed high homology to group 10 allergen of storage mites, group 10 allergen of house dust mites and *P. ovis* tropomyosin. Group 10 house dust mite allergen is conserved and cross-reactive with tropomyosin of species such as shellfish and other arthropods [26]. Furthermore, patients who are allergic to house dust mites show clinical symptoms when eating shellfish [27]. The prediction of cross-reactivity indicated a highly significant sequence match of SsTm with allergens of other mites. Thus, tropomyosin is not a specific allergen for Sarcoptic mites and may have high cross-reactivity with tropomysin of other mites and invertebrates [28].

In this study, SsTm was localized by immunohistochemistry in the mouthparts, muscle and legs of scabies mites, as similarly observed in *P. ovis*[19]. As an actin-binding protein, tropomyosin can stabilize actin fibers and plays an important role in development and activities of vertebrates [11]. Moreover, the mouthparts and legs of mites are important organs for feeding and burrowing. Hence, it is possible that the function of tropomyosin is related to the survival activities of *S. scabiei.*

Many studies have identified tropomyosin as a highly allergenic protein from parasites, such as nematodes [29], trematodes [30] and mites [31], and it induces Th2-IgE mediated responses in sheep infested with the sheep scab mite [18]. Furthermore, specific IgG and IgE antibody levels have been shown to be dramatically increased in rabbits and goats exhibiting signs of sarcoptic mange [31,32]. In addition, IgE is also an important antibody in the host defense against scabies mites [32].

Although the whole mite extracts have been used in studies of *S.scabiei* vaccination trials recently, abundant mites are difficult to acquire owing to the lack of an *in vitro* culture system [6]. Furthermore, tropomyosin has been demonstrated to effectively induce protective responses against other parasites such as the filarial nematode *A. viteae* as mentioned above. The troponin I-like protein of *Haemaphysalis longicornis*, which has been considered as a potential vaccine candidate antigen, may be identical to tropomyosin [33]. Hence, we chose the recombinant protein as a potential candidate antigen. In the previous studies, we stopped the experiment four weeks after infestation because scabies mange of rabbits may cause devastating effects of fitness in the short-term [34]. Furthermore, lesion areas could hardly be measured if mites transferred throughout the body of the rabbit such as limbs and head. In this study, immunization of rabbits with recombinant SsTm in the present study did not provide significant protection against *S. scabiei* challenge, although a high level of specific IgG and a decrease in lesion areas were elicited. Findings of two previous vaccination studies [24,35] with insoluble and soluble mite protein fractions in goats infested with *S. scabiei* var. *ovis* revealed that the responses did not have any protective value. We obtained a similar result to the latter study in which vaccination with the soluble protein fraction induced high levels of scabies-specific IgG but failed to induce a significant level of specific IgE. Vaccination with soluble recombinant SsTm in this current study likewise did not generate evident levels of IgE in rabbits. Conversely, goats that received repeated mite challenge have been shown to develop strong IgG and IgE antibody responses [23]. Another report showed that extremely high total IgE and IgG levels were induced in the serum of crusted scabies patients [36]. In the current study, a significant rise in specific IgG antibody was detected in vaccinated animals compared to the control animals, but the total IgE antibody level was not obviously elevated within four weeks after the challenge. Thus, low IgE levels may either indicate that SsTm truly had little effect or that the infection time period was insufficient for the influence of this protein to be observed.

IgG responses against whole *S. scabiei* antigen extracts have been using to diagnose sarcoptic mange in pigs [37] and also to evaluate protective effects via serum IgG and IgE responses in a vaccination trial against *S. scabiei*. In another previous study, it was suggested that the mite densities after challenge in animals vaccinated with fresh mite extracts were considerably lower than those in control animals, although skin lesions were still obvious in all goats [38]. Hence, our results indicate that antibodies induced by SsTm may not be the effective response needed for protection. Another similar vaccination study of Ssag1 and Ssag2 in rabbits showed no protective effect, although the immunized rabbits did not exhibit the typical crust characteristics [39]. Nevertheless, differences in lesion characteristics were not obvious between the vaccinated and unvaccinated groups in our study. It seems that the allergenicity of SsTm was too low to induce adequate protective immunity or perhaps subtle structural changes of recombinant SsTm led to a loss of allergenicity and ability to generate a protective immune response. Due to the lack of immunization studies against *S. scabiei* with recombinant mite proteins, it is unclear why tropomyosin has been identified as an allergenic protein but not when isolated from mites.

The immune response mechanism of scabies mites remains unclear despite the attention this field has received by many researchers. Cell-mediated immunity has been shown to be principally involved but antibodies are also relevant immune responses induced by immunization against scabies mites [40]. Unlike other ectoparasites, which live on the skin surface of the host, scabies mites thrive in the epidermis of the host and feed on epidermal proteins and host plasma. It is possible that the low level of protective immunoglobulin is ingested into the gut or that tropomyosin is not the target of protective immunity in *S. scabiei*. Multiple studies on the specific mechanisms of immune evasion in scabies mites have shown a multigene family of inactivated scabies mite serine proteases (SMIPPs) to be responsible for the adaptation to parasitism in the epidermal burrows, and these proteins may also have an influence on protective efficacy [41], [42].

## Conclusions

In summary, we have characterized an allergenic protein SsTm from *S. scabiei* by demonstrating its localization in muscle tissues, mouthparts and legs, and implicated its potentially significant functional role in *S. scabiei*. Although, no significant protective effect was detected against mite challenge in rabbits vaccinated with recombinant SsTm, multiple vaccination studies have suggested that tropomyosin should be evaluated for its vaccine potential. This study established a foundation for further research on recombinant vaccines in scabies mite. Clearly, future studies should focus on development of more effective methods to enhance the protective efficacy of recombinant vaccines against sarcoptic mange.

## Abbreviations

BSA, Bovine Serum Albumin; BCIP, 5-Bromo-4-Chloro-3'-Indolyphosphate; ELISA, Enzyme-Linked Immunosorbent Assay; HRP, Horseradish Peroxidase; IPTG, Isopropyl-Beta-D-Thiogalactopyranoside; SsTm, Sarcoptes scabiei Tropomyosin; NBT, Nitro-Blue Tetrazolium; PBS, Phosphate Buffered Saline; SDAP, Interleukin-10; TBS, Tris-Buffered Saline; TBST, Tris-Buffered Saline And Tween 20; TMB, 3,3’,5,5’-TetraMethylBenzidine.

## Competing interests

The authors declare that they have no competing interests.

## Authors' contributions

RZ and QJ participated in the design of the study, manuscript writing and performed the statistical analysis; WZ participated in vaccination trials and discussion; YR and XN participated in the collection of mite samples and data; XW carried out the molecular genetic studies; XG prepared figures and tables; SW participated in sequence alignment, XP prepared figures and helped to draft the manuscript; SL provided the support of experimental animals; GY participated in the design of study and have given final approval of the version; All authors read and approved the final manuscript.

## References

[B1] McCarthy J Kemp D Walton S Currie B Scabies: more than just an irritation Postgrad Med J 2004 80 945 382 387 10.1136/pgmj.2003.014563 15254301 PMC1743057

[B2] Hengge UR Currie BJ Jager G Lupi O Schwartz RA Scabies: a ubiquitous neglected skin disease Lancet Infect Dis 2006 6 769 779 10.1016/S1473-3099(06)70654-5 17123897

[B3] Taplin D Meinking TL Chen JA Sanchez R Comparison of crotamiton 10% cream (Eurax) and permethrin 5% cream (Elimite) for the treatment of scabies in children Pediatr Dermatol 1990 7 67 73 10.1111/j.1525-1470.1990.tb01078.x 2188239

[B4] Pence DB Ueckermann E Sarcoptic mange in wildlife Rev Sci Tech 2002 21 385 398 11974622

[B5] Millán J Casáis R Delibes-Mateos M Calvete C Rouco C Castro F Colomar V Casas-Díaz E Ramírez E Moreno S Widespread exposure to Sarcoptes scabiei in wild European rabbits (Oryctolagus cuniculus) in Spain Vet Parasitol 2011 183 (3-4) 323 329 21852039 10.1016/j.vetpar.2011.07.046

[B6] Walton SF Holt DC Currie BJ Kemp DJ Scabies: new future for a neglected disease Adv Parasitol 2004 57 309 376 15504541 10.1016/S0065-308X(04)57005-7

[B7] Rehbein S Visser M Winter R Trommer B Matthes HF Maciel A Marley S Productivity effects of bovine mange and control with ivermectin Vet Parasitol 2003 114 267 284 10.1016/S0304-4017(03)00140-7 12809753

[B8] Pasay C Rothwell J Mounsey K Kelly A Hutchinson B Miezler A McCarthy J An exploratory study to assess the activity of the acarine growth inhibitor, fluazuron, against Sarcoptes scabei infestation in pigs Parasit Vectors 2012 5 1 40 10.1186/1756-3305-5-40 22336283 PMC3298804

[B9] Heukelbach J Feldmeier H Scabies Lancet 2006 367 9524 1767 1774 10.1016/S0140-6736(06)68772-2 16731272

[B10] Alasaad S Walton S Rossi L Bornstein S Abu-Madi M Soriguer RC Fitzgerald S Zhu XQ Zimmermann W Ugbomoiko US Sarcoptes-World Molecular Network (Sarcoptes-WMN): integrating research on scabies Int J Infect Dis 2011 15 5 e294 e297 10.1016/j.ijid.2011.01.012 21454116

[B11] Perry SV Vertebrate tropomyosin: distribution, properties and function J Muscle Res Cell Motil 2001 22 1 5 49 10.1023/A:1010303732441 11563548

[B12] Reese G Ayuso R Lehrer SB Tropomyosin: An Invertebrate Pan-allergen Int Arch Allergy Immunol 1999 119 4 247 258 10.1159/000024201 10474029

[B13] Asturias JA Gómez-Bayón N Arilla MC Martínez A Palacios R Sánchez-Gascón F Martínez J Molecular characterization of American cockroach tropomyosin (Periplaneta americana allergen 7), a cross-reactive allergen J Immunol 1999 162 7 4342 4348 10201967

[B14] Nisbet A Huntley J Mackellar A Sparks N McDevitt R A house dust mite allergen homologue from poultry red mite Dermanyssus gallinae (De Geer)* Parasite Immunol 2006 28 8 401 405 10.1111/j.1365-3024.2006.00862.x 16879312

[B15] Witteman A Akkerdaas J Van Leeuwen J Van Der Zee J Aalberse R Identification of a cross-reactive allergen (presumably tropomyosin) in shrimp, mite and insects Int Arch Allergy Immunol 1994 105 1 56 61 10.1159/000236803 8086829

[B16] Chu KH Wong SH Leung PSC Tropomyosin is the major mollusk allergen: reverse transcriptase polymerase chain reaction, expression and IgE reactivity Mar Biotechnol 2000 2 5 499 509 11246417 10.1007/s101260000035

[B17] Hartmann S Adam R Marti T Kirsten C Seidinger S Lucius R A 41-kDa antigen of the rodent filaria Acanthocheilonema viteae with homologies to tropomyosin induces host-protective immune responses Parasitol Res 1997 83 4 390 393 10.1007/s004360050269 9134565

[B18] Huntley J Machell J Nisbet A Van den Broek A Chua K Cheong N Hales B Thomas W Identification of tropomyosin, paramyosin and apolipophorin/vitellogenin as three major allergens of the sheep scab mite, Psoroptes ovis Parasite Immunol 2004 26 335 342 10.1111/j.0141-9838.2004.00717.x 15679630

[B19] Nisbet A MacKellar A Wright H Brennan G Chua K Cheong N Thomas J Huntley J Molecular characterization, expression and localization of tropomyosin and paramyosin immunodominant allergens from sheep scab mites (Psoroptes ovis) Parasitology 2006 133 4 515 523 10.1017/S0031182006000631 16817997

[B20] Mattsson J Ljunggren E Bergstrom K Paramyosin from the parasitic mite Sarcoptes scabiei: cDNA cloning and heterologous expression Parasitology 2001 122 05 555 562 11393829 10.1017/s0031182001007648

[B21] Chen WJ Niu DS Zhang XY Chen ML Cui H Wei WJ Wen BH Chen XR Recombinant 56-kilodalton major outer membrane protein antigen of Orientia tsutsugamushi Shanxi and its antigenicity Infect Immun 2003 71 8 4772 4779 10.1128/IAI.71.8.4772-4779.2003 12874360 PMC166048

[B22] Liddell S Knox D Extracellular and cytoplasmic Cu/Zn superoxide dismutases from Haemonchus contortus Parasitology 1998 116 4 383 394 10.1017/S0031182098002418 9585940

[B23] Rodríguez-Cadenas F Carbajal-González M Fregeneda-Grandes J Aller-Gancedo J Rojo-Vázquez F Clinical evaluation and antibody responses in sheep after primary and secondary experimental challenges with the mange mite Sarcoptes scabiei var. ovis Ve Immunol Immunopathol 2009 133 2–4 109 116 10.1016/j.vetimm.2009.07.004 19783311

[B24] Tarigan S Huntley JF Failure to protect goats following vaccination with soluble proteins of Sarcoptes scabiei: evidence for a role for IgE antibody in protection Vet Parasitol 2005 133 1 101 109 10.1016/j.vetpar.2005.03.044 16118041

[B25] Tamura K Dudley J Nei M Kumar S MEGA4: molecular evolutionary genetics analysis (MEGA) software version 4.0 Mol Biol Evol 2007 24 8 1596 1599 10.1093/molbev/msm092 17488738

[B26] Arlian LG Morgan MS Vyszenski-Moher DAL Sharra D Cross-reactivity between storage and dust mites and between mites and shrimp Exp App Acarol 2009 47 2 159 172 10.1007/s10493-008-9199-x 18850281

[B27] Becker S Gröger M Canis M Pfrogner E Kramer MF Tropomyosin sensitization in house dust mite allergic patients Eur Arch Otorhinolaryngol 2011 269 (4) 1 6 22081096 10.1007/s00405-011-1826-1

[B28] Reese G Jeoung BJ Daul CB Lehrer SB Characterization of recombinant shrimp allergen Pen a 1 (tropomyosin) Int Arch Allergy Immunol 1997 113 1–3 240 242 9130534 10.1159/000237558

[B29] Hartmann S Sereda MJ Sollwedel A Kalinna B Lucius R A nematode allergen elicits protection against challenge infection under specific conditions Vaccine 2006 24 17 3581 3590 10.1016/j.vaccine.2006.01.064 16504347

[B30] Newport GR McKerrow J Hedstrom R Petitt M McGarrigle L Barr P Agabian N Cloning of the proteinase that facilitates infection by schistosome parasites J Biol Chem 1988 263 26 13179 13184 3166457

[B31] Thomas WR Smith WA Hales BJ The allergenic specificities of the house dust mite Chang Gung Med J 2004 27 8 563 569 15553602

[B32] Walton S The immunology of susceptibility and resistance to scabies Parasite Immunol 2010 32 8 532 540 20626808 10.1111/j.1365-3024.2010.01218.x

[B33] You MJ Immunization effect of recombinant P27/30 protein expressed in Escherichia coli against the hard tick Haemaphysalis longicornis (Acari: Ixodidae) in rabbits Korean J Parasitol 2004 42 4 195 200 10.3347/kjp.2004.42.4.195 15591837 PMC2717385

[B34] Millán J First description of sarcoptic mange in wild European rabbit (Oryctolagus cuniculus) Eur J Wildl Res 2010 56 3 455 457 10.1007/s10344-009-0347-3

[B35] TARIGAN S Protective value of immune responses developed in goats vaccinated with insoluble proteins from Sarcoptes scabiei JITV 2005 10 2 118 126

[B36] Walton SF Beroukas D Roberts-homson P Currie B New insights into disease pathogenesis in crusted (Norwegian) scabies: the skin immune response in crusted scabies Br J Dermatol 2008 158 6 1247 1255 10.1111/j.1365-2133.2008.08541.x 18422789

[B37] Kessler E Matthes HF Schein E Wendt M Detection of antibodies in sera of weaned pigs after contact infection with Sarcoptes scabiei var. suis and after treatment with an antiparasitic agent by three different indirect ELISAs Vet Parasitol 2003 114 1 63 73 10.1016/S0304-4017(03)00098-0 12732467

[B38] TARIGAN S Vaccination of Goats with Fresh Extract from Sarcoptes scabiei Confers Partial Protective Immunity JITV 2006 11 2 144 150

[B39] Fischer K Holt DC Harumal P Currie BJ Walton SF Kemp DJ Generation and characterization of cDNA clones from Sarcoptes scabiei var. hominis for an expressed sequence tag library: identification of homologues of house dust mite allergens Am J Trop Med Hyg 2003 68 1 61 12556150

[B40] Şenol M Özerol İ Özerol E Şaşmaz S Turan F Soytürk D Serum Immunoglobulin and Complement Levels in Scabies J Inonu University Medical Faculty 2010 4 1 37 39

[B41] Holt DC Fischer K Allen GE Wilson D Wilson P Slade R Currie BJ Walton SF Kemp DJ Mechanisms for a novel immune evasion strategy in the scabies mite Sarcoptes scabiei: a multigene family of inactivated serine proteases J Invest Dermatol 2003 121 6 1419 1424 10.1046/j.1523-1747.2003.12621.x 14675192

[B42] Lambris JD Ricklin D Geisbrecht BV Complement evasion by human pathogens Nat Rev Microbiol 2008 6 2 132 142 10.1038/nrmicro1824 18197169 PMC2814840

